# Case Report: Persistent severe thrombocytopenia after adjuvant ado-trastuzumab emtansine with sequential bone marrow findings and romiplostim support

**DOI:** 10.3389/fonc.2026.1859304

**Published:** 2026-06-17

**Authors:** Dan Qiao, Lei Nie, Shuang Liu, Hai-bin Ding

**Affiliations:** Department of Medical Oncology, Shaanxi Provincial Cancer Hospital, Xi’an, Shaanxi, China

**Keywords:** ado-trastuzumab emtansine, breast cancer, case report, romiplostim, thrombocytopenia

## Abstract

Ado-trastuzumab emtansine (T-DM1) remains an established adjuvant option for patients with human epidermal growth factor receptor 2 (HER2)-positive early breast cancer and residual invasive disease after neoadjuvant treatment. Severe, persistent thrombocytopenia deviating from the usual cyclic pattern is uncommon and clinically challenging. We report a 50-year-old woman who developed sustained grade 4 thrombocytopenia after three cycles of adjuvant T-DM1, following prior platinum-based chemotherapy and during adjuvant radiotherapy. The thrombocytopenia was refractory to recombinant human thrombopoietin and hetrombopag. Additional evaluations found no evidence of splenomegaly, peripheral platelet destruction, clonal hematologic disease, or a localized radiation effect on the sampled marrow. Sequential marrow evaluations supported megakaryocytic maturation impairment, with initial depletion followed by quantitative recovery but persistently few platelet-producing forms, without dysplasia or fibrosis. Platelet counts repeatedly declined after romiplostim interruption and recovered after reintroduction, allowing long-term stabilization with a threshold-based intermittent regimen. Following multidisciplinary discussion, further anti-HER2 therapy was withheld because of the severely compromised thrombopoietic reserve. As adjuvant antibody-drug conjugate (ADC) strategies continue to evolve, this case suggests that severe non-cyclic thrombocytopenia may occur early during HER2-directed ADC therapy. It supports the value of sequential marrow assessment in selected refractory cases and provides clinical context for individualized romiplostim support in prolonged thrombocytopenia.

## Introduction

Ado-trastuzumab emtansine (T-DM1) is a human epidermal growth factor receptor 2 (HER2)-targeted antibody-drug conjugate (ADC) composed of trastuzumab linked to the microtubule inhibitor DM1 (1). In patients with HER2-positive early breast cancer and residual invasive disease after neoadjuvant therapy, adjuvant T-DM1 became an established post-neoadjuvant treatment based on the KATHERINE trial, which showed improved invasive disease-free survival compared with trastuzumab, with later follow-up confirming an overall survival benefit (2–4). More recently, trastuzumab deruxtecan (T-DXd) showed superior invasive disease-free survival compared with T-DM1 in the post-neoadjuvant setting for high-risk residual HER2-positive early breast cancer, reflecting ongoing changes in adjuvant HER2-directed ADC therapy (5).

Thrombocytopenia is a well-recognized dose-limiting toxicity of T-DM1. In the adjuvant KATHERINE trial, thrombocytopenia occurred in 28.5% of patients receiving T-DM1, and grade ≥3 thrombocytopenia occurred in 5.7% (2). In the metastatic EMILIA trial, thrombocytopenia occurred in 31.1% of patients receiving T-DM1, and grade 3–4 thrombocytopenia occurred in 14.5% (6). Higher rates have been reported in Chinese cohorts and other Asian populations, suggesting clinically relevant variability in susceptibility (7–9). In most patients, platelet counts decrease after dosing, reach a nadir approximately 7 to 10 days later, and recover before the next scheduled cycle (10).

The mechanisms of T-DM1-associated thrombocytopenia are not fully defined. Preclinical studies suggest that T-DM1 and its maytansinoid payload can impair megakaryocyte differentiation, proplatelet formation, and platelet production through microtubule-dependent pathways (11).

These findings are consistent with broader evidence that ADC-related toxicities may arise from target-dependent and target-independent uptake, intracellular payload release, and payload-specific injury in susceptible normal cells (12). Persistent, severe thrombocytopenia that deviates from the expected cyclic pattern remains uncommon, and its evaluation and management are not well established. We describe a patient who developed sustained grade 4 thrombocytopenia after three cycles of adjuvant T-DM1, with sequential marrow findings, limited platelet recovery during recombinant human thrombopoietin (rhTPO)/hetrombopag treatment, and long-term stabilization during intermittent romiplostim support.

## Case description

### Baseline characteristics and initial treatment

A 50-year-old postmenopausal woman with amenorrhea for 2 years presented on March 15, 2023, with a right breast mass. Physical examination revealed a firm, ill-defined, poorly mobile mass measuring approximately 3 × 2.5 cm in the upper-outer quadrant of the right breast, with palpable right axillary lymph nodes. Core biopsy confirmed invasive carcinoma with ER 70%, PR 70%, HER2 3+, and Ki-67 40%. Clinical staging was cT2N1M0, stage IIB, according to the AJCC 8th edition, with no evidence of distant metastasis. The patient reported no family history of breast or other hereditary cancers. She had no known history of liver disease, autoimmune disease, bleeding diathesis, or venous or arterial thrombosis. She was not receiving antiplatelet agents, anticoagulants, nonsteroidal anti-inflammatory drugs, or herbal or dietary supplements. From March 21 to July 12, 2023, she received six 3-weekly cycles of nab-paclitaxel plus trastuzumab/pertuzumab and carboplatin (area under the curve, 5), without dose modification. Platelet counts remained within the normal range during neoadjuvant therapy, although grade 2 neutropenia occurred (CTCAE v5.0). Imaging demonstrated a partial response, with a 68.5% reduction by RECIST v1.1.

On August 3, 2023, she underwent a right modified radical mastectomy. Pathology showed residual invasive carcinoma of no special type (NST), grade II, measuring approximately 1.5 cm, with ypT1cN0M0 disease, stage IA, according to the AJCC 8th edition. Immunohistochemistry on the surgical specimen demonstrated ER 90%, PR 70%, HER2 3+, and Ki-67 30%. T-DM1 was initiated at 200 mg (3.6 mg/kg) on August 29, September 19, and October 16, 2023; baseline blood counts before T-DM1 were normal. Adjuvant intensity-modulated radiotherapy (IMRT) was delivered from September 18 to October 18, 2023, to the chest wall, including the mastectomy bed, and the ipsilateral supraclavicular and axillary level II–III nodal regions. The planned dose was 50 Gy in 25 fractions, but radiotherapy was discontinued after 18 fractions, with a cumulative dose of 36 Gy, because of severe thrombocytopenia.

### Onset of thrombocytopenia and first bone marrow evaluation

After cycle 1 of T-DM1, grade 1 thrombocytopenia occurred on day 9 (September 7, 2023; nadir 87×10^9^/L) and resolved spontaneously. A similar transient decline occurred after cycle 2 (day 7; September 26, 2023; nadir 94×10^9^/L).

Three days after cycle 3 (October 19, 2023), the platelet count abruptly decreased to 29×10^9^/L, prompting interruption of radiotherapy. rhTPO was initiated on October 20 at 15,000 units daily, but the platelet count further declined to 14×10^9^/L by October 22, and hetrombopag 7.5 mg daily was added. A transient increase to 57×10^9^/L was observed on October 24, followed by progressive decline. On November 10, 2023, the platelet count reached 6×10^9^/L, accompanied by extensive ecchymoses and persistent gingival bleeding, requiring readmission and platelet transfusion.

**Figure 1:**
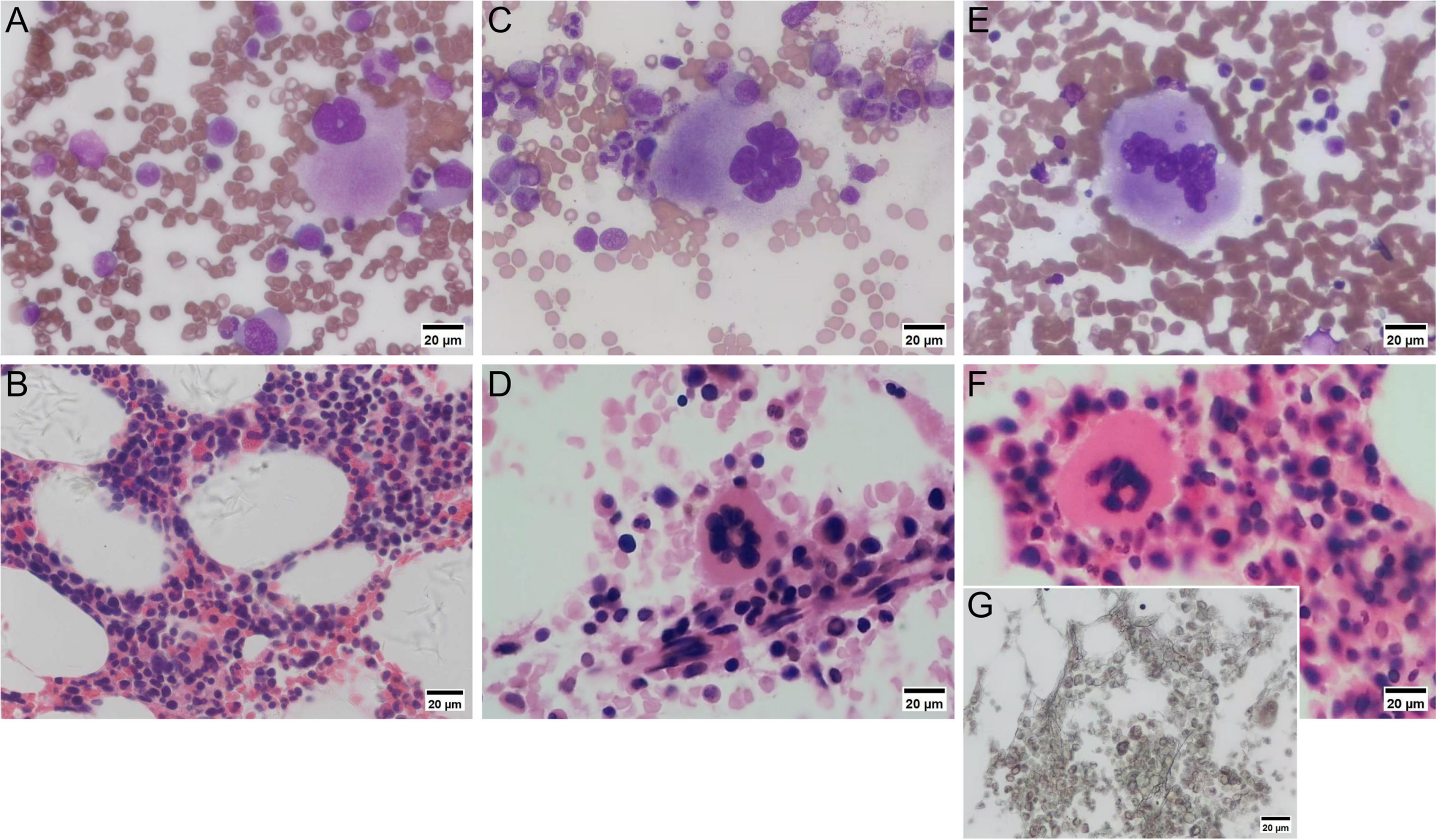
Representative bone marrow aspirate and biopsy findings from three sequential evaluations (original magnification, ×400 for all panels; scale bars = 20 μm). **(A, B)** November 14, 2023: Aspirate smear **(A)** shows a single granular megakaryocyte against a background of marked megakaryocytic depletion. The corresponding core biopsy **(B)** demonstrates active erythroid and myeloid hematopoiesis but no megakaryocytes in this representative field. **(C, D)** June 12, 2024: Aspirate smear **(C)** shows a representative granular megakaryocyte. The corresponding biopsy **(D)** demonstrates a megakaryocyte with a mature lobulated nucleus. **(E, F)** August 7, 2025: Aspirate smear **(E)** shows a granular megakaryocyte. The corresponding biopsy **(F)** shows a megakaryocyte with retained nuclear lobulation. **(G)** Gomori reticulin staining from August 7, 2025, showing no reticulin fibrosis (MF-0). Together with the quantitative smear findings and pathology reports described in the text, these representative fields illustrate the transition from initial megakaryocytic depletion to quantitative recovery with persistent impairment in platelet-producing forms. Wright–Giemsa staining was used for **(A, C, E)**; hematoxylin and eosin (H&E) staining for **(B, D, F)**; and Gomori reticulin staining for **(G)**.

Bone marrow aspiration and biopsy were performed on November 14, 2023, from the posterior iliac crest, outside the radiation field. Smear review showed markedly reduced megakaryocytes, with only seven identified across the entire smear. The corresponding biopsy confirmed active marrow hematopoiesis with a markedly reduced number of megakaryocytes and no evidence of abnormal cellular infiltration or reticulin fiber proliferation. The pathology report interpreted the marrow as markedly hypoplastic with reduced megakaryocyte representation. Representative fields are shown in **Figures 1A, B**. A peripheral blood smear showed no schistocytes. On November 10, a complete blood count revealed red blood cell (RBC) 4.2×10^12^/L, white blood cell (WBC) 5.4×10^9^/L, and hemoglobin 125 g/L. Coagulation parameters, liver and renal function tests, and lactate dehydrogenase were within normal limits. Viral screening for hepatitis B virus (HBV), hepatitis C virus (HCV), and human immunodeficiency virus (HIV) was negative, as were platelet-associated antibodies. Abdominal ultrasonography of the liver, gallbladder, pancreas, and spleen showed no hepatobiliary abnormality, portal vein dilatation, cirrhosis, or splenomegaly.

### Refractory course and switch to romiplostim

After the first marrow evaluation, thrombocytopenia persisted despite prior rhTPO and hetrombopag treatment. Hetrombopag was discontinued because the platelet increase was not sustained, and romiplostim was initiated on November 17, 2023, at 180 μg weekly (3 μg/kg). Because the platelet count remained below 25×10^9^/L, the dose was increased to 250 μg weekly (4.2 μg/kg) during week 2 and maintained at that level. During the initial weekly phase, platelet counts increased to 36–59×10^9^/L.

After the dose on December 18, 2023, the patient discontinued romiplostim. The platelet count subsequently declined to 15×10^9^/L on January 8, 2024, accompanied by epistaxis and petechiae, and platelet transfusion was required. Romiplostim was resumed, after which the platelet count recovered to above 60×10^9^/L.

**Figure 2:**
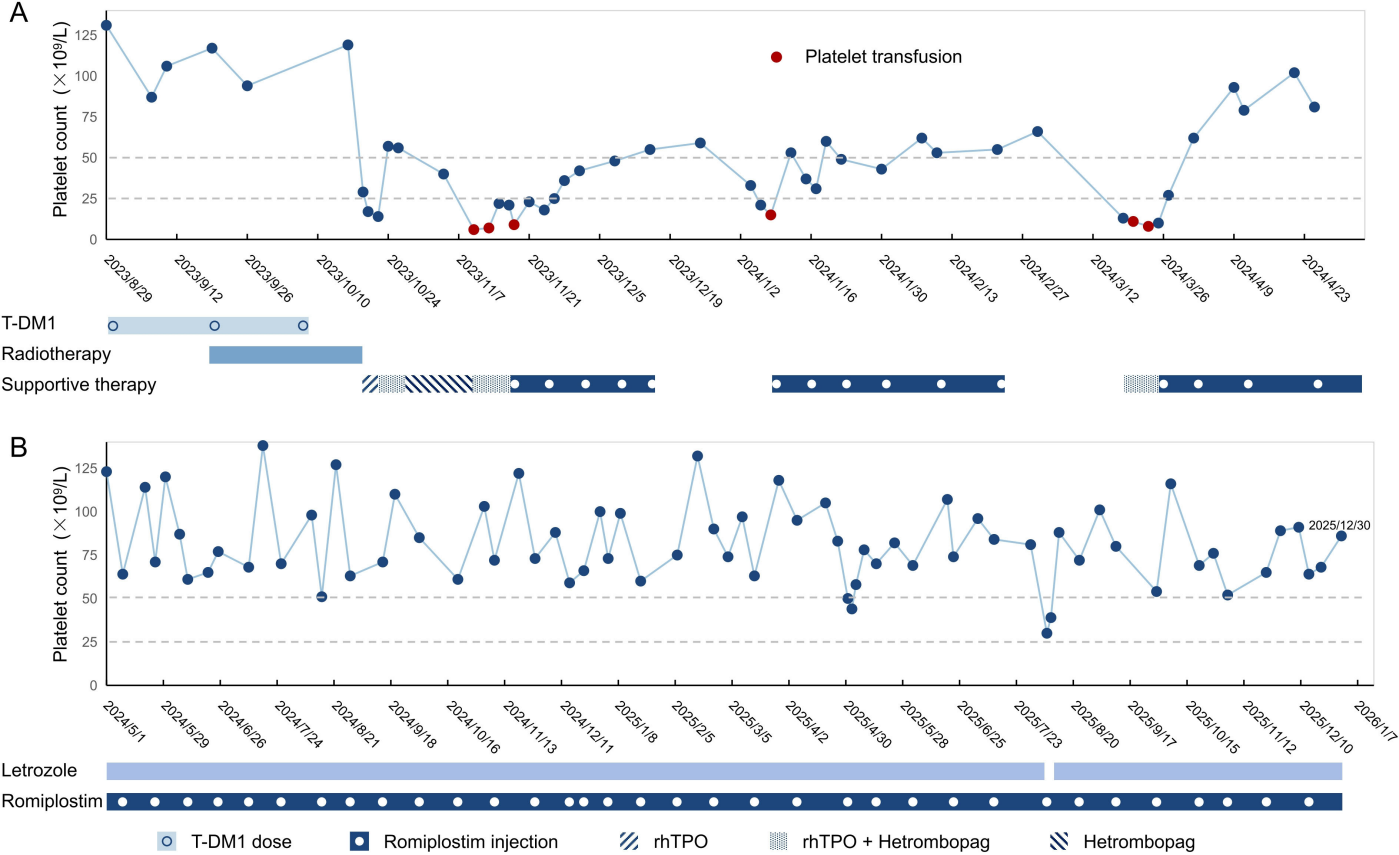
Longitudinal platelet counts and treatment timeline. **(A)** Acute phase (Aug 29, 2023–Apr 30, 2024), showing severe thrombocytopenia during rhTPO/hetrombopag treatment and platelet recovery after romiplostim reintroduction. **(B)** Maintenance phase (May 1, 2024–Dec 30, 2025), showing platelet stabilization during intermittent romiplostim support.

In March 2024, romiplostim was interrupted again from March 1 to March 25 because of drug unavailability. During this interval, a 7-day course of rhTPO combined with hetrombopag was given, but platelet counts had decreased to 8–13×10^9^/L by March 18, requiring additional transfusions. Romiplostim was reintroduced at 250 μg on March 25, followed by gradual platelet recovery. From April 2024 onward, platelet counts were maintained predominantly within 62–102×10^9^/L, allowing progressive extension of the dosing interval to 15–22 days (**Figure 2A**).

### Longitudinal assessment and long-term outcome

Adjuvant endocrine therapy with letrozole was initiated on May 1, 2024, after platelet counts had stabilized above 60×10^9^/L with romiplostim support. A second bone marrow aspiration and biopsy were performed on June 12, 2024, from the posterior iliac crest, outside the radiation field. The marrow showed active hematopoiesis with quantitative recovery of megakaryocytes. Smear review identified 64 megakaryocytes per slide; among 20 classified cells, 16 were granular forms and 4 were platelet-producing forms. The corresponding biopsy demonstrated normocellular marrow, with megakaryocytes predominantly showing lobulated nuclei. Based on the smear findings and biopsy assessment, the pathology report interpreted the marrow as proliferative with megakaryocytic maturation impairment. Representative fields are shown in **Figures 1C, D**. Repeat assessments in June 2024 showed negative platelet-associated antibodies, no schistocytes on peripheral blood smear, and no splenomegaly on abdominal ultrasonography. During the maintenance phase, romiplostim dosing intervals were gradually extended to approximately every 2–3 weeks, while platelet counts were generally maintained within 50–138×10^9^/L (**Figure 2B**).

On July 30, 2025, the patient discontinued romiplostim. The platelet count declined from 84×10^9^/L on July 12 to 30×10^9^/L on August 7, 2025. Romiplostim was re-administered on August 7, and a third marrow evaluation was performed the same day. The platelet count increased to 39×10^9^/L by August 9 and to 88×10^9^/L by August 13. The third marrow evaluation, again performed from the posterior iliac crest, showed active hematopoiesis. Smear review identified 82 megakaryocytes per slide; among 20 classified cells, 18 were granular forms and 2 were platelet-producing forms. The corresponding biopsy demonstrated normocellular marrow, with megakaryocytes predominantly showing lobulated nuclei. Based on the combined smear and biopsy findings, the pathology report again interpreted the marrow as proliferative with megakaryocytic maturation impairment. Gomori reticulin staining was negative, consistent with myelofibrosis grade 0 (MF-0), and flow cytometry showed no evidence of clonal hematologic malignancy. Representative fields are shown in **Figures 1E–G**. Repeat autoimmune and platelet antibody panels were negative. Peripheral blood smears showed no schistocytes, and repeat abdominal ultrasonography showed no splenomegaly.

The thrombocytopenia was considered most likely related to prior T-DM1 exposure, with prior platinum-based chemotherapy and overlapping adjuvant radiotherapy considered potential contributing factors. Switching to non-ADC HER2-directed therapy, including trastuzumab with or without pertuzumab, was discussed during multidisciplinary consultation. Given the recurrent grade 4 thrombocytopenia, bleeding events, and continued need for romiplostim support, the multidisciplinary team recommended withholding further HER2-directed therapy at that time. Radiotherapy was not resumed. At the last follow-up on December 30, 2025, platelet counts remained within a clinically safe range with intermittent romiplostim, without major bleeding or thrombotic events, and there was no evidence of disease recurrence.

## Discussion

This case differs from the usual cyclic pattern of T-DM1-associated thrombocytopenia, in which platelet counts typically decline after dosing and recover before the next cycle (13). The patient developed abrupt grade 4 thrombocytopenia after the third adjuvant cycle, followed by a persistent course that required permanent discontinuation of T-DM1. The platelet count declined during interruptions of romiplostim and increased after reintroduction, consistent with ongoing thrombopoietic impairment rather than a short-lived dosing effect. Non-ADC HER2-directed therapy, including trastuzumab with or without pertuzumab, was discussed. This decision was not based on a presumed major thrombocytopenic effect of trastuzumab itself, but on the patient’s recurrent grade 4 thrombocytopenia, bleeding events, and continued dependence on romiplostim, which indicated a narrow safety margin for any additional systemic therapy at that time.

A structured evaluation was performed to distinguish impaired platelet production from peripheral consumption, as recommended for persistent or severe cancer therapy-associated thrombocytopenia (14). Peripheral blood smears showed no schistocytes, coagulation parameters and biochemical tests were within normal limits, viral and autoimmune testing, including platelet-associated antibodies, remained negative, and abdominal ultrasonography showed no splenomegaly or hepatobiliary findings suggestive of hypersplenism. These findings did not support thrombotic microangiopathy, secondary immune thrombocytopenia, hypersplenism, or clinically overt hepatic dysfunction as the main explanation.

The sequential marrow evaluations were central to identifying impaired platelet production. The first examination showed marked megakaryocytic depletion, whereas later aspirate smears showed quantitative recovery of megakaryocytes with persistently few platelet-producing forms. The corresponding biopsies showed normocellular marrow with lobulated megakaryocytes and no dysplasia or reticulin fibrosis. The interpretation of megakaryocytic maturation impairment was based on the complete marrow evaluation, including smear counts, cell classification, biopsy findings, and pathology reports, rather than the representative images alone. This pattern is compatible with impaired platelet production and proplatelet formation (15). The marrow findings, however, do not establish a single causative mechanism.

The severity and persistence of thrombocytopenia in this patient appeared multifactorial. T-DM1 exposure was considered the most likely clinical trigger, while prior platinum-based chemotherapy and overlapping adjuvant radiotherapy may have reduced hematopoietic reserve and increased susceptibility to clinically significant thrombocytopenia (7, 16, 17). The marrow evaluations were performed from the posterior iliac crest, outside the radiation field, making a purely localized radiation effect less likely. A contribution from radiotherapy-related hematopoietic stress, however, cannot be excluded (18, 19). The DM1 payload of T-DM1 may impair megakaryocyte differentiation and proplatelet formation through microtubule disruption (11, 20, 21). This mechanism is compatible with the observed megakaryocytic abnormalities, but remains biologically plausible rather than definitive, given the single-patient design and the absence of direct mechanistic assays.

The platelet pattern during thrombopoietic support is clinically relevant but should be interpreted cautiously. In this patient, platelet increases after rhTPO and hetrombopag were not sustained, whereas romiplostim interruption and reintroduction were temporally associated with platelet decline and recovery. Fang Wang et al. reported that, in early HER2-positive breast cancer, recovery from T-DM1-induced thrombocytopenia may take longer with rhTPO than with thrombopoietin receptor agonists (TPO-RAs), which provides clinical context for this observation (16). Evidence from chemotherapy-induced thrombocytopenia also supports an association between romiplostim or other TPO-RAs and platelet recovery in patients with severe thrombocytopenia, including real-world data and systematic analyses (22, 23). Romiplostim and hetrombopag differ pharmacologically, with romiplostim acting as a peptibody agonist of the thrombopoietin receptor and hetrombopag acting as an oral non-peptide agonist at a distinct receptor site (24, 25). These differences provide mechanistic context but do not establish a drug-specific advantage in a single patient.

Evidence specific to ADC-associated persistent thrombocytopenia remains limited. Current guidance for chemotherapy-induced thrombocytopenia emphasizes close platelet monitoring, platelet transfusion when bleeding risk is high, and consideration of TPO-RAs when persistent or recurrent thrombocytopenia compromises cancer therapy (26). Additional reviews and meta-analytic data suggest that longer-term TPO-RA support may be feasible in selected patients with chemotherapy-induced thrombocytopenia and may improve platelet recovery or reduce treatment delays (27, 28). These data should be extrapolated cautiously to refractory ADC-associated thrombocytopenia, given differences in mechanism, timing, and clinical course between ADC-related thrombocytopenia and conventional chemotherapy-induced thrombocytopenia. In this patient, intermittent, threshold-based romiplostim support was feasible and well tolerated, with no reticulin fibrosis on follow-up marrow assessment and platelet counts maintained within a clinically safe range.

The clinical context for post-neoadjuvant HER2-directed ADC therapy has changed with the reporting of DESTINY-Breast05. DESTINY-Breast05 showed superior invasive disease-free survival with T-DXd compared with T-DM1 in patients with high-risk residual HER2-positive early breast cancer (5). The toxicity profiles of T-DM1 and T-DXd are not interchangeable. Thrombocytopenia is a characteristic concern with T-DM1 (2, 6). In contrast, T-DXd requires close attention to interstitial lung disease/pneumonitis, as well as gastrointestinal toxicity and myelosuppression (29, 30). Pharmacovigilance data comparing trastuzumab, T-DXd, and T-DM1 further support distinct adverse-event patterns across HER2-directed therapies (31). This case remains relevant in the ADC setting because it illustrates the need for early recognition and individualized supportive management of persistent, non-cyclic hematologic toxicity.

This report has several limitations. It describes a single patient, and pharmacokinetic testing, anti-drug antibody testing, receptor signaling assays, and pharmacogenomic analyses were not performed. The relative contributions of T-DM1, prior platinum-based chemotherapy, overlapping adjuvant radiotherapy, and time-dependent marrow recovery cannot be separated with certainty. The platelet changes observed during different thrombopoietic interventions should also be interpreted cautiously, because timing effects, supportive care, and spontaneous marrow recovery may have contributed to the platelet trajectory. The mechanistic interpretation and the observed platelet pattern during thrombopoietic support should therefore be considered hypothesis-generating.

## Conclusion

This case describes severe, persistent, non-cyclic thrombocytopenia after adjuvant T-DM1, with sequential marrow assessment showing early megakaryocytic depletion followed by quantitative recovery with persistently few platelet-producing forms. The temporal association between romiplostim interruption/reintroduction and platelet changes is consistent with ongoing thrombopoietic impairment, but a drug-specific advantage over rhTPO or hetrombopag cannot be inferred from a single case. Intermittent, threshold-based romiplostim support may be considered in selected refractory cases.

## Patient perspective

During my treatment, my platelet counts were carefully monitored. Since starting romiplostim, my platelet levels have stabilized, giving me more confidence in continuing therapy. I am grateful for the support and guidance from the medical team throughout this process.

## Data Availability

The original contributions presented in the study are included in the article/**Supplementary Material**. Further inquiries can be directed to the corresponding author.
